# Identification of a novel *RASD1* somatic mutation in a *USP8*-mutated corticotroph adenoma

**DOI:** 10.1101/mcs.a001602

**Published:** 2017-05

**Authors:** Andrew V. Uzilov, Khadeen C. Cheesman, Marc Y. Fink, Leah C. Newman, Chetanya Pandya, Yelena Lalazar, Marco Hefti, Mary Fowkes, Gintaras Deikus, Chun Yee Lau, Aye S. Moe, Yayoi Kinoshita, Yumi Kasai, Micol Zweig, Arpeta Gupta, Daniela Starcevic, Milind Mahajan, Eric E. Schadt, Kalmon D. Post, Michael J. Donovan, Robert Sebra, Rong Chen, Eliza B. Geer

**Affiliations:** 1 Department of Genetics and Genomic Sciences and Icahn Institute for Genomics and Multiscale Biology, Icahn School of Medicine at Mount Sinai, New York, New York 10029, USA;; 2 Division of Endocrinology, Diabetes, and Bone Disease, Department of Medicine, Icahn School of Medicine at Mount Sinai, New York, New York 10029, USA;; 3 Department of Pathology, Icahn School of Medicine at Mount Sinai, New York, New York 10029, USA;; 4 Department of Neurosurgery, Icahn School of Medicine at Mount Sinai, New York, New York 10029, USA;; 5 Multidisciplinary Pituitary and Skull Base Tumor Center, Memorial Sloan Kettering, New York, New York 10065, USA

**Keywords:** adrenocorticotropic hormone excess, increased circulating ACTH level, increased circulating cortisol level, neoplasm of the anterior pituitary, neoplasm of the endocrine system, pituitary corticotropic cell adenoma

## Abstract

Cushing's disease (CD) is caused by pituitary corticotroph adenomas that secrete excess adrenocorticotropic hormone (ACTH). In these tumors, somatic mutations in the gene *USP8* have been identified as recurrent and pathogenic and are the sole known molecular driver for CD. Although other somatic mutations were reported in these studies, their contribution to the pathogenesis of CD remains unexplored. No molecular drivers have been established for a large proportion of CD cases and tumor heterogeneity has not yet been investigated using genomics methods. Also, even in *USP8*-mutant tumors, a possibility may exist of additional contributing mutations, following a paradigm from other neoplasm types where multiple somatic alterations contribute to neoplastic transformation. The current study utilizes whole-exome discovery sequencing on the Illumina platform, followed by targeted amplicon-validation sequencing on the Pacific Biosciences platform, to interrogate the somatic mutation landscape in a corticotroph adenoma resected from a CD patient. In this *USP8*-mutated tumor, we identified an interesting somatic mutation in the gene *RASD1*, which is a component of the corticotropin-releasing hormone receptor signaling system. This finding may provide insight into a novel mechanism involving loss of feedback control to the corticotropin-releasing hormone receptor and subsequent deregulation of ACTH production in corticotroph tumors.

## INTRODUCTION

Cushing's syndrome is caused by chronic exposure to elevated glucocorticoids via exogenous and endogenous sources. The typical clinical features of Cushing's syndrome are related to hypercortolism and include accumulation of central fat, moon facies, neuromuscular weakness, osteoporosis or bone fractures, metabolic complications, and mood changes. It is associated with increased morbidity and mortality especially due to cardiovascular disease (Lacroix et al. 2015; Sharma et al. 2015). Adrenocorticotropic hormone (ACTH)-dependent Cushing's accounts for 80% of endogenous cases, and among these, pituitary corticotroph adenomas are the most common cause (Lacroix et al. 2015). This is known as Cushing's disease (CD). Corticotroph adenomas account for ∼10% of pituitary adenomas and the vast majority of them are benign. The incidence of CD ranges from 1.2 to 2.4 per million population per year in Europe and up to 8 per million population per year in the United States (Etxabe and Vazquez 1994; Lindholm et al. 2001; Feelders et al. 2012; Broder et al. 2015).

Genetic factors involved in corticotroph tumorigenesis are largely unknown. ACTH-secreting adenomas due to germline mutations can rarely arise in the context of familial disorders, such as multiple endocrine neoplasia type 1 (MEN1), familial isolated pituitary adenomas (associated with aryl-hydrocarbon receptor-interacting protein [gene *AIP*] mutations), and MEN4 (associated with cyclin-dependent kinase inhibitors) (Dworakowska and Grossman 2012; Perez-Rivas and Reincke 2016). Rare somatic mutations in the *TP53* gene (Kawashima et al. 2009) and in the glucocorticoid receptor (gene *NR3C1*) and related proteins have also been reported (Lacroix et al. 2015; Ma et al. 2015; Reincke et al. 2015).

The identification of recurrent somatic mutations that lead to CD has been elusive until the recent discovery of somatic mutations in the ubiquitin-specific peptidase 8 gene (*USP8*) in 35%–62% of CD-causing corticotroph adenomas (Perez-Rivas and Reincke 2016). The *USP8* gene encodes an enzyme with deubiquitinase activity. To date, 22 different *USP8* mutations have been identified in 129 ACTH-secreting adenomas from 271 patients across three studies (Ma et al. 2015; Perez-Rivas et al. 2015; Reincke et al. 2015; for review, see Perez-Rivas and Reincke 2016), with confirmation of *USP8* mutation prevalence in later studies (Hayashi et al. 2016; Song et al. 2016). All of these mutations were located in exon 14 in a mutation hotspot region that overlaps with the sequence that codes for the 14-3-3 binding motif, which is highly conserved among different species. This mutation constitutively activates *USP8*, leading to enhanced recycling of the epidermal growth factor receptor (gene *EGFR*) to the plasma membrane, resulting in sustained signaling and increased ACTH synthesis (Perez-Rivas and Reincke 2016).

The distinction between a tumor's monoclonal origin (in which all tumor cells are descendants of a single cell in which a driver mutation occurred) versus polyclonal origin (in which tumor cells are a mixture of multiple clonal expansions, possibly containing different driver mutations) is important to understanding the disease mechanism. The clonal origin of a tumor can be determined by X-Chromosome inactivation analysis in female patients with heterozygous alleles at various X-linked loci (Levy 2001). Previous studies using this class of techniques have shown that all (Biller et al. 1992; Gicquel et al. 1992) or most (Herman et al. 1990; Schulte et al. 1991) corticotroph adenomas are monoclonal. However, arguments for polyclonality in a nonnegligible fraction of corticotroph and other pituitary adenomas have been proposed (Clayton et al. 2000; Levy 2000, 2001; Clayton and Farrell 2001, 2004). Analysis of allelic fractions of somatic mutations and germline variants in tumor genomic sequencing data can potentially shed light on the genetic heterogeneity and clonal origin of corticotroph adenomas, but such analysis has not been carried out in previous studies where such data were available (Ma et al. 2015; Reincke et al. 2015).

Genome- and exome-wide analyses on DNA from tumors and patient-matched normal controls have been instrumental in identifying driver genes in many neoplasm types. In this study, we performed whole-exome sequencing (WES) on a tumor sample from a patient with CD and on blood samples from the patient and her two healthy sisters (identical triplets; data from the two healthy sisters was not used in this case report). We have identified a novel mutation in the GTP-binding site of the gene *RASD1* that we hypothesize contributes to the pathogenesis of CD in this patient because of the involvement of *RASD1* in regulation of ACTH production by glucocorticoid feedback. Additionally, based on the allelic fractions of the mutations, these tumor cells exhibit either subclones or polyclonal origin. These findings challenge the current model that corticotroph adenomas are genetically homogeneous.

## RESULTS

### Clinical Presentation and Family History

**Figure 1.**
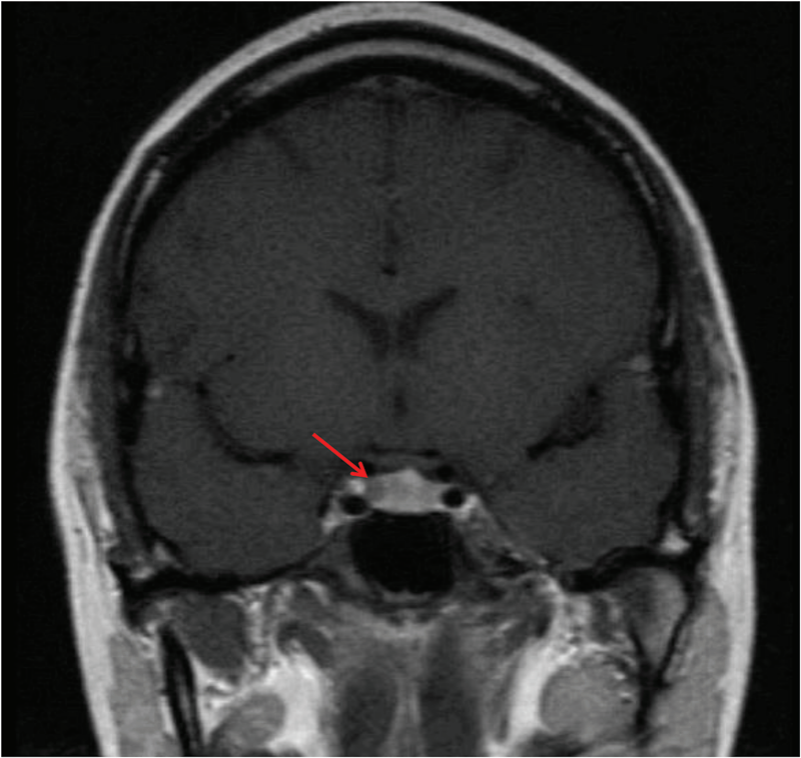
Pituitary magnetic resonance imaging (MRI) coronal image confirmed a right-sided sellar hypointensity consistent with a 4-mm pituitary adenoma.

**Figure 2.**
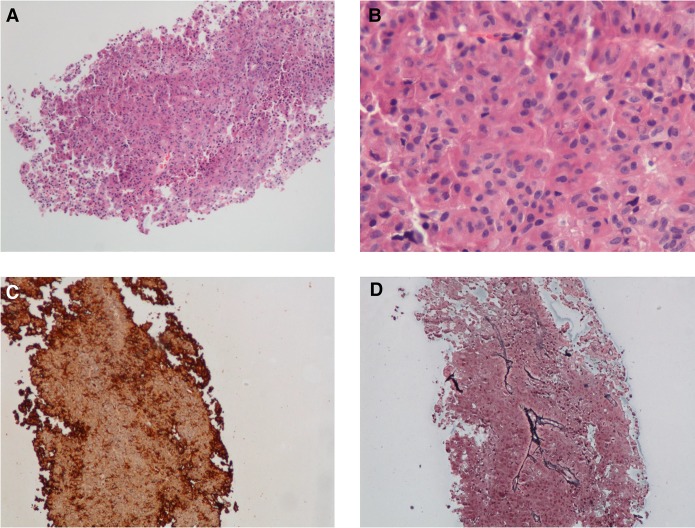
Histologic sections revealed a monotonous tumor composed of medium-sized cells with finely stippled chromatin (*A*,*B*). Tumor cells were strongly positive for adenocorticotropic hormone (ACTH) by immunohistochemistry (*C*), whereas a reticulin stain (*D*) showed effacement of the fibrovascular septae. (*A*) Hematoxylin and eosin (H&E) 100×; (*B*) H&E 400×; (*C*) ACTH immunostain, 100×; (*D*) reticulin, 100×.

A 32-year-old woman with no known family history of pituitary diseases, who was one of identical triplet sisters, presented with a history of recently diagnosed type 2 diabetes mellitus and weight gain, easy bruising, and subjective plethora. On review of systems she endorsed occasional acne, moodiness before her menstrual period, and chronic insomnia. She denied neuromuscular weakness, significant mood changes, difficulty concentrating, or hirsutism. Evaluation confirmed CD: 24-h urinary free cortisol (UFC) levels were 154 and 90.4 µg (nl < 50 µg); midnight salivary cortisol (MSC) levels were 0.118, 0.142, and 0.917 µg/dl (nl < 0.112 µg/dl); serum cortisol was 4.1 µg/dl after 8 mg dexamethasone (nl < 1.8 µg/dl); random morning plasma ACTH level was 50 pg/ml; and pituitary magnetic resonance imaging (MRI) showed a clearly defined right-sided 4-mm lesion (Fig. 1). She was also found to have dyslipidemia and fatty liver on MRI of the abdomen. She was diagnosed with CD and underwent transnasal transsphenoidal adenomectomy without complication. Immunohistochemistry confirmed a corticotroph adenoma (Fig. 2) with a low MIB-1 index. Pathologic examination of tissue slices estimated 40%–50% tumor cellularity.

### Genomic Analyses

**Figure 3.**
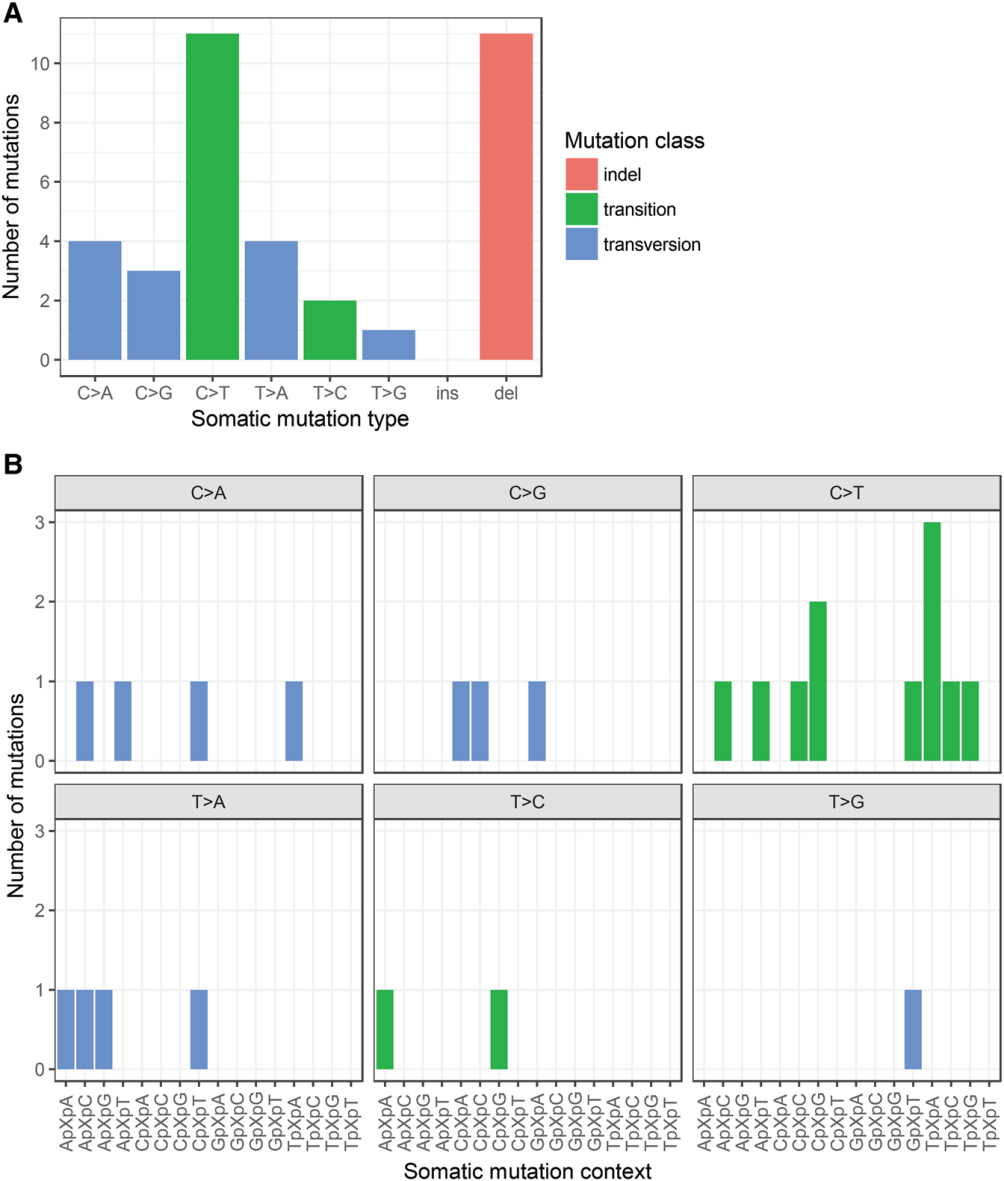
(*A*) Distribution of the 36 somatic mutation calls passing manual review by type and class. All passing calls were included regardless of predicted impact (i.e., synonymous, intronic, and intergenic calls are included). For single-nucleotide variants (SNVs), type is given by the pyrimidine of the mutated base pair as per previous conventions (Alexandrov et al. 2013). (*B*) SNVs from *A* were further categorized into 96 trinucleotide classes defined by the immediately flanking bases (as previously described in Alexandrov et al. 2013).

WES of the blood-derived normal DNA and formalin-fixed paraffin-embedded (FFPE)-derived tumor DNA from the patient was carried out on the Illumina HiSeq 2500 platform, yielding a mean sequencing depth of 145× and 315×, respectively, that was usable for variant calling (Table 1). Germline (constitutional) variants and somatic mutations were called; supporting binary alignment (BAM) read alignments for each somatic mutation were manually reviewed in Integrative Genomics Viewer (IGV) (Robinson et al. 2011; Thorvaldsdóttir et al. 2013), yielding 36 passing calls whose class, type, and trinucleotide context are depicted in Figure 3 (the variant genomic coordinates are provided as Supplemental File 1). C>T transitions were the most common single-nucleotide variant (SNV) type, as is common in many cancer mutation signatures (Alexandrov et al. 2013); although importantly Alexandrov et al. (2013) did not include mutations outside protein-coding exons, which are included in Fig. 3. Notably, the mutations were dominated by deletions, only two of which were in protein-coding regions. No insertions were observed (Table 2).

**Table 1.** Sequencing and alignment statistics for Illumina whole-exome sequencing (WES) of the patient's normal/tumor pair

Sample	Number of clusters yielding “pass filter” read pairs (PF_READS/2)	Mean usable sequencing depth	Percent target bases with >30× usable sequencing depth	Percent usable bases out of all “pass filter” read bases (PCT_USABLE_BASES_ON_TARGET)	Percent duplication (PERCENT_DUPLICATION)
Normal	77 × 10^6^	145×	96	74	10
Tumor	335 × 10^6^	315×	98	37	54

Data as given by the Genome Analysis Toolkit (GATK) v3.2 (McKenna et al. 2010; DePristo et al. 2011; Van der Auwera et al. 2013) and Picard (http://broadinstitute.github.io/picard) pipelines.

Picard terminology is as defined on https://broadinstitute.github.io/picard/picard-metric-definitions.html, with the Picard output field name given in parentheses. “Duplication” includes both optical/sequencing and polymerase chain reaction (PCR) duplicates according to Picard and is computed on the entire genome (i.e., not just in exome target regions). “Usable” read bases are bases aligned to exome-target regions and not in duplicate reads.

**Table 2.** Somatic mutations in the patient's tumor that were predicted to alter the protein sequence (single-nucleotide variants [SNVs]: missense, nonsense, affecting canonical splice site; indels: affecting coding exon, affecting canonical splice site), ordered by decreasing tumor allelic fraction of the alternate (nonreference) allele in the Illumina whole-exome sequencing (WES) data (ILMN)

									Tumor						Normal				
									Alt allele fraction (%)		ILMN		PB		ILMN		PB		
Gene symbol	Gene name	Gene ID	Chromosome	Mutation in DNA	Mutation in protein	Variant type	Predicted effect	dbSNP ID	ILMN	PB	Total reads	Alt allele reads	Total reads	Alt allele reads	Total reads	Alt alele reads	Total reads	Alt alele reads	Comments
*AIG1*	Androgen- induced 1	51390	6	NC_000006.11:g.143656205A>T	p.(Met239Leu)	SNV	missense_variant		26	21*	338	88	8,182*	1,730*	148	0	953	0	Protein is predicted to change only in transcript isoform ENST00000275235 (intronic in all RefSeq and UniProt isoforms)
*ATP8A2*	ATPase phospholipid-transporting 8A2	51761	13	NC_000013.10:g.26138117A>T	p.(Asp434Val), p.(Asp474Val)	SNV	missense_variant		24	23*	649	158	8,158*	1,874*	333	0	709	1	
*USP8*	Ubiquitin-specific peptidase 8	9101	15	NC_000015.9:g.50782647C>G	p.(Pro720Arg), p.(Pro614Arg)	SNV	missense_variant	rs672601311	22	20*	333	72	7,044*	1,417*	141	0	694	0	Pathogenic mechanism previously described (Ma et al. 2015; Reincke et al. 2015); ClinVar accession RCV000149420.1
*FRYL*	FRY-like transcription coactivator	285527	4	NC_000004.11:g.48529993G>C	p.(Pro2379Ala)	SNV	missense_variant		20	5	81	16	757	38	29	0	523	0	
*PRPF18*	Pre-mRNA processing factor 18	8559	10	NC_000010.10:g.13642266A>G	p.(Gln56Arg)	SNV	missense_variant		19	15	471	89	1,288	190	169	0	1,090	1	
*LGI3*	Leucine-rich repeat LGI family member 3	203190	8	NC_000008.10:g.22006350G>C	p.(Gln324Glu)	SNV	missense_variant		9	9	235	20	411	38	90	0	464	0	
*MINK1*	Misshapen like kinase 1	50488	17	NC_000017.10:g.4795451AGAG>A	p.(Arg671del)	deletion	inframe_deletion		8	8	310	26	4,602	367	131	1	459	2	
*PPFIBP2*	PPFIA binding protein 2	8495	11	NC_000011.9:g.7661089C>A	p.(Leu455Met)	SNV	missense_variant		7	2	154	11	518	9	120	0	563	0	
*SLFN12*	Schlafen family member 12	55106	17	NC_000017.10:g.33749940CA>C	p.(Leu36Argfs*6)	deletion	frameshift_variant		6	nd	501	28	nd	nd	385	1	nd	nd	Did not attempt PB validation
*PPEF1*	Protein phos phatase with EF-hand domain 1	5475	X	NC_000023.10:g.18800487T>A	p.(Tyr243*)	SNV	stop_gained		5	7	152	7	667	45	87	0	415	0	Region undergoes copy loss based on CNA analysis; gene has an intronic somatic SNV (NC_000023.10:g.18752019G>A) with alt allele fraction of 17%
*MMP26*	Matrix metallo peptidase 26	56547	11	NC_000011.9:g.5012679G>A	p.(Gly183Glu)	SNV	missense_variant		4	3*	185	8	6,025*	188*	117	0	148	0	
*RASD1*	Ras-related dexameth asone induced 1	51655	17	NC_000017.10:g.17399395T>A	p.(Lys34Met)	SNV	missense_variant		3	3*	406	12	32,529*	923*	175	0	1,790	1	A different somatic mutation (p.K34R) has been observed at this position (COSMIC database mutation ID: COSM5385794)
*DCHS2*	Dachsous cad herin-related 2	54798	4	NC_000004.11:g.155219314G>A	p.(Ser1596Leu)	SNV	missense_variant	rs747828053	3	20	557	19	576	118	430	0	588	0	

Read depth statistics are also shown for validation of mutations by targeted amplicon sequencing on PacBio (PB). Some amplicons yielded inconclusive evidence during the first PB sequencing run, and thus starred (*) data are from a second run where multiplexing was adjusted to yield higher depth for the given amplicons. Gene symbols and names are from HUGO Gene Nomenclature Committee (HGNC) (Gray et al. 2015) (retrieved 2016-05-25). Gene IDs are from the National Center for Biotechnology Information (NCBI) Gene (http://www.ncbi.nlm.nih.gov/gene, retrieved 2016-05-25). Mutations are given according to Human Genome Variation Society (HGVS) nomenclature (den Dunnen et al. 2016) version 15.11. Unless otherwise noted, amino acid numbering is from all canonical isoforms based on review of all Reference Sequencing Database (RefSeq) (Pruitt et al. 2014) and UniProtKB (The UniProt Consortium et al. 2015) isoforms at the given genomic location in the UCSC Genome Browser (Kent et al. 2002) (retrieved 2016-05-25). Predicted amino acid change and effect are from SnpEff version 4.0b (Cingolani et al. 2012) using Sequence Ontology terms (Cunningham et al. 2015). For ILMN data, read counts for total reads and reads supporting alternate allele are taken directly from the respective variant caller MuTect (Cibulskis et al. 2013) for SNVs and Varscan2 (Koboldt et al. 2012) for indels.

dbSNP, Database for Short Genetic Variations; COSMIC, Catalogue of Somatic Mutations in Cancer.

After variant annotation, 13 of 36 somatic mutation calls were predicted to alter the amino acid sequence of a protein isoform (Table 2). This count of 13 protein-altering mutations is consistent with the low counts previously reported in other WES studies of corticotroph adenomas: median 7, range 3–23 (Reincke et al. 2015) and median 5, range 1–9 (Ma et al. 2015). The difference with Ma et al. (2015) can be explained by the higher sequencing depth in our study and differences in variant-calling procedure described in Ma et al. (2015); calls with allelic fraction of <20% were discarded, which would have eliminated most of our calls (Table 2). Validation was carried out on 12 of the 13 mutations via targeted amplicon sequencing on a second next-generation sequencing (NGS) platform (Pacific Biosciences RSII, PacBio), confirming 100% of the attempted mutations as somatic (present in tumor, absent in normal) and also obtaining similar allelic fractions to the original Illumina-based calls. Following the heuristic from Alexandrov et al. (2013) that WES interrogates ∼30 megabases (Mb) of protein-coding exons in the human genome, we obtained a somatic mutation rate of 0.43 mutations/Mb for protein-altering mutations and 0.53 mutations/Mb for mutations in coding exons (13 protein-altering; three synonymous). When the latter is compared with somatic mutation rates across many cancer types in Alexandrov et al. (2013), the somatic mutation rate in our patient was low, a finding consistent with previous WES studies of corticotroph adenomas (Ma et al. 2015; Reincke et al. 2015).

The allelic fractions of somatic mutations ranged from 3% to 26%. Under the hypothesis that one of these mutations initiates or precedes neoplastic transformation of a single cell that then undergoes clonal expansion, the tumor purity of the sequenced specimen may therefore be <52%, consistent with the estimate of 40%–50% from pathologic examination. Although the presence of copy-number alterations (CNAs) can confound such an estimate, we did not observe aneuploidy or CNA events that confound the allelic fractions in Table 2 (using the SAAS-CNV tool [Zhang and Hao 2015] as before [Uzilov et al. 2016]; data not shown).

We cross-referenced our somatic mutation calls with the Catalogue of Somatic Mutations in Cancer (COSMIC) database (Forbes et al. 2015) to determine whether any had previously been observed in a tumor; no exact matches were found and one approximate match is noted in Table 2. We also cross-referenced these calls with ClinVar (Landrum et al. 2016), as some variants are known to occur as both somatic driver mutations and germline variants in inherited/familial neoplasm syndromes. We then reviewed the gene annotations in the list of 13 protein-altering mutations in the context of potential involvement in the molecular pathways implicated in CD or in corticotroph tissues in general. Notably, our patient's adenoma had the *USP8* p.P720R mutation that has previously been described as involved in the pathogenesis of CD (Ma et al. 2015; Reincke et al. 2015), occurring in 35%–62% of CD-causing corticotroph adenomas (Perez-Rivas and Reincke 2016); this was also the sole mutation identified in the ClinVar cross-reference. The high allelic fraction of this mutation (20%–22%, depending on sequencing platform; third highest on the list) is consistent with the hypothesis that it is a driver mutation present early in the clonal expansion of the tumor.

**Figure 4.**
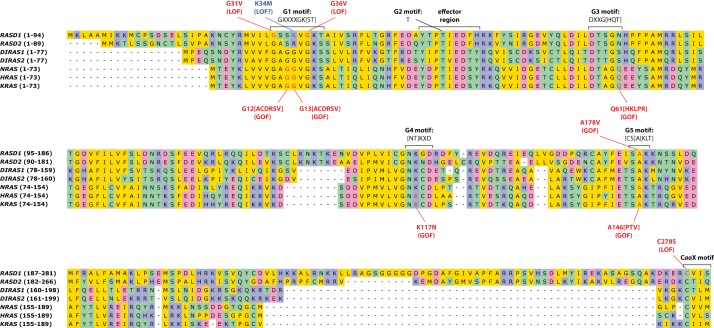
Multiple sequence alignment of select proteins in the Ras family of small monomeric GTPases to which *RASD1* belongs (Wennerberg et al. 2005). Indicated motifs (taken from Wennerberg et al. 2005 and Bourne et al. 1991), and then verified via UniProt (The UniProt Consortium et al. 2015; retrieved 2016-09-24): GDP/GTP-binding G-box motifs (G1–G5); effector region; C*aa*X amino-terminal motif that undergoes posttranslational modification (*a* denotes any aliphatic amino acid). *RASD2*, encoding the protein Rhes, is shown because it is the closest human homolog to *RASD1* (63% protein sequence identity); the two form the RASD subfamily that is distinct from other Ras family proteins (<45% protein sequence identity). *DIRAS1* and *DIRAS2* are shown because they are the next closest homologs to RASD family proteins. *NRAS*, *HRAS*, and *KRAS* are shown because they are well-characterized oncogenes. Functional impact of the *RASD1* mutations (in red) has been demonstrated experimentally: p.G31V (Cismowski et al. 1999, 2000; Vaidyanathan et al. 2004), p.G36V (Cismowski et al. 1999), p.A178V (Graham et al. 2001), and p.C278S (Graham et al. 2001; Vaidyanathan et al. 2004). Mutations in *NRAS*, *HRAS*, and *KRAS* (in red) are widely known oncogenic mutations and are also recurrent somatic mutations across multiple neoplasm types in COSMIC (Forbes et al. 2015) (accessed 2016-09-25), except for *HRAS* amino acid A146 (no mutations of any type in COSMIC, although p.A146V results in constitutive activation [Feig and Cooper 1988] and may be germline pathogenic in Costello syndrome, ClinVar accession RCV000013445.18), and *NRAS* amino acid K117 (no mutations of any type in COSMIC and no published evidence on any K117 mutation). Amino acid ranges are given in parentheses next to the gene symbols. Amino acids are color-coded according to biochemical class (yellow, nonpolar; green, polar; blue, basic; pink, acidic). For positions where one of several amino acids is possible, the possibilities are given in brackets. X, any amino acid; LOF, loss of function; GOF, gain of function.

A novel mutation, p.K34M, in the GTP-binding region of *RASD1* was identified in this tumor, at an allelic fraction (3%) indicative of a subclone with respect to cells containing *USP8* p.P720R. *RASD1* was originally discovered as an inducible gene in dexamethasone-stimulated AtT-20 mouse corticotroph cells (Kemppainen 1998). Based on this connection to the cell type under study, further computational analysis of the functional significance of this mutation was conducted. Alignment of several related small GTPases, including the well-studied oncogenes *KRAS*, *NRAS*, and *HRAS*, revealed that p.K34M is found within the G1 motif and is likely involved in binding to GTP (Fig. 4). However, the precise contacts between the enzyme and substrate are not clear given that the G1 motif diverges in the RASD family from the other small GTPases for which substrate-bound crystal structures are available. Other genes in Table 2 were reviewed for possible connections to CD or corticotroph biology but were not judged to have a direct connection like *USP8* or *RASD1*; hence, these two genes are the focus of this report.

### Treatment Outcomes

Consistent with successful removal of the patient's ACTH-secreting tumor, her postoperative day 1 serum cortisol level was 3.4 µg/dl. She was discharged on physiologic oral hydrocortisone replacement and subsequently tapered off after 4 mo. Following discontinuation of oral hydrocortisone, UFC, 8 mg dexamethasone suppressed serum cortisol, and MSC concentrations were all within the normal range, consistent with CD remission. CD symptoms and comorbidities, including blood glucose levels, also improved. She remains in remission 3.3 yr after surgery.

## DISCUSSION

### Tumor Genetic Heterogeneity Models

**Figure 5.**
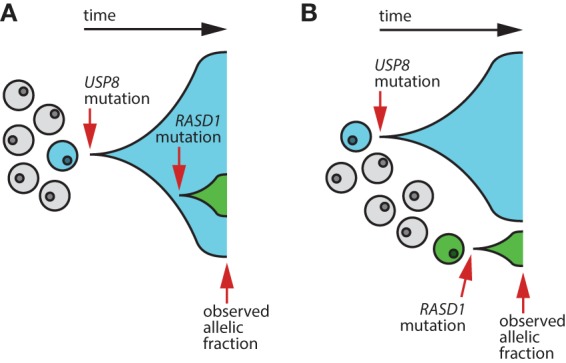
Two models that explain the observed allelic fractions of the *USP8* and *RASD1* somatic mutations. (*A*) Mutation *RASD1* p.K34M occurs in a single cell derived from the clonal expansion of *USP8*-mutant cells. (*B*) Mutation *RASD1* p.K34M occurs in an independent, *USP8*-wild-type cell and undergoes clonal expansion separate from the *USP8*-mutant clonal expansion.

The current case identifies a novel *RASD1* mutation in a *USP8*-positive corticotroph adenoma. The different allelic fractions between the *USP8* and *RASD1* somatic mutations in the studied tumor cells suggest that this ACTH-secreting tumor is genetically heterogeneous. Two models for heterogeneity are proposed as follows. In model A (Fig. 5A), the *USP8* and *RASD1* mutations may be synergistic, with the *USP8* mutation occurring early in tumorigenesis, leading to abnormal proliferation of ACTH-secreting cells. At a later time point in the pathogenesis of the tumor, one of these *USP8*-mutant cells acquires a *RASD1* mutation, resulting in a subclone of *RASD1*-mutant/*USP8*-mutant cells. In model B (Fig. 5B), the *USP8* and *RASD1* mutations are mutually exclusive, giving rise to subclones of cells with different mutation combinations (i.e., *USP8*-mutant/*RASD1*-wild type vs. *USP8*-wild type/*RASD1*-mutant). In both models, the tumor is genetically heterogeneous. Our hypothesis is that under both these models, *RASD1* is a contributor to cell proliferation and ACTH secretion, but occurs in a small subpopulation of the tumor cells. Although these findings do not clearly distinguish between monoclonal versus polyclonal origin of the tumor, they nevertheless indicate that the tumor is genetically heterogeneous and suggest further studies into the interplay between multiple possible drivers.

### Rationale for *RASD1* as a Contributor to Pathogenesis

**Figure 6.**
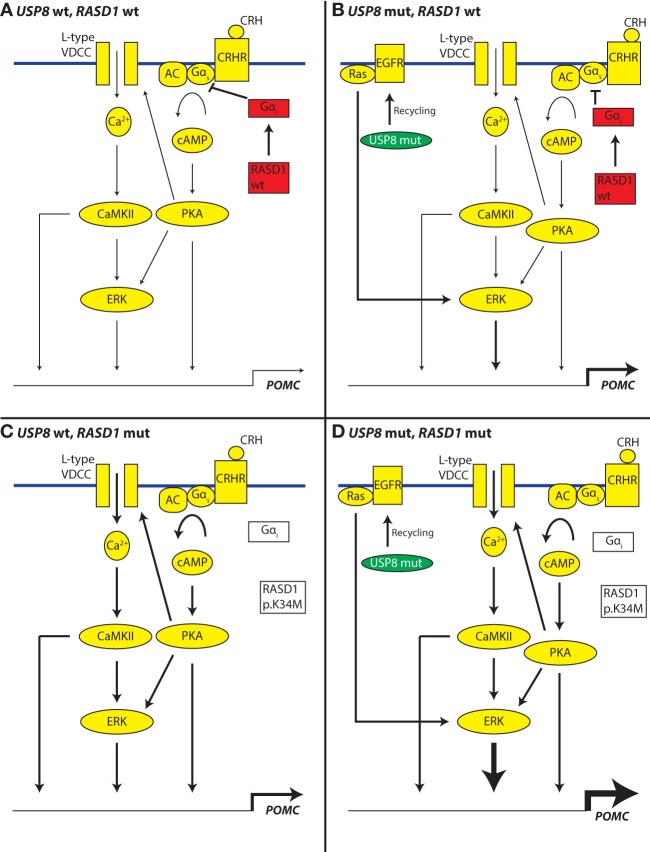
Hypothesized altered feedback control of the *POMC* gene promoter in cells having *USP8* or *RASD1* mutations (mut) versus wild type (wt). Contributing flux through pathway components and the effect of *POMC* (proopiomelanocortin gene) transcription are shown in cartoon form as small/medium/large arrow thicknesses. Pathway diagram is based on Jenks (2009). (*A*) Signaling through the corticotropin-releasing hormone receptor (CRHR) in the context of intact feedback inhibition as indicated by active *RASD1* and Gα_i_ (red) allows for coordinated biosynthesis and secretion of ACTH. (*B*) *USP8* mutation (green) allows for enhanced activity of the EGFR-recycling apparatus and thereby triggers stronger positive regulation of adenocorticotropic hormone (ACTH) production. (*C*) Signaling through the CRHR in the context of disrupted feedback inhibition as indicated by *RASD1* and Gα_i_ (white and disconnected from Gα_s_) may allow for dysregulated and increased secretion of ACTH. (*D*) Signaling schematic in the context of both *USP8* and *RASD1* mutation, showing the possible additive or synergistic effects downstream from extracellular signal-regulated kinase (ERK). AC, adenylyl cyclase; Ca^2+^, calcium; CaMKII, calmodulin-dependent protein kinase II; cAMP, cyclic adenosine monophosphate; PKA, protein kinase A; VDCC, voltage-dependent calcium channel.

This study identified a mutation in *RASD1* that may alter binding to GTP on the basis of *RASD1* structural homology with well-studied, oncogenic small GTPases *KRAS*, *NRAS*, and *HRAS*. It is appealing to speculate that *RASD1* p.K34M will have a reduced affinity to GTP and will therefore be less active (decreased capacity to interact with downstream proteins) versus wild type. However, because of the low allelic fraction (presumed subclonal nature) of this mutation, we cannot conclusively determine whether the mutation is homozygous or heterozygous. Several studies have defined a role for *RASD1* in inhibition of Gα_s_ signaling (Graham et al. 2001, 2004). This may occur through an interaction with Gα_i_ (Cismowski et al. 2000)_._ Normal feedback regulation within the hypothalamic–pituitary–adrenal axis involves glucocorticoid induction of genes associated with suppression of corticotropin-releasing hormone receptor (CRHR) signaling. *RASD1* was identified by its virtue of being strongly induced by dexamethasone in mouse corticotroph cell lines and pituitaries (Kemppainen 1998; Tu and Wu 1999; Brogan et al. 2001). Taken together, *RASD1* may be a transcriptionally inducible negative regulator of CRHR-Gα_s_ signaling in corticotrophs. In cells with nonfunctional *RASD1*, this loop is no longer intact and CRHR signaling may become insensitive to negative feedback from glucocorticoids, thereby allowing for continued ACTH secretion. This alteration within corticotroph signaling and regulation may occur within the population of *USP8* mutant cells or in a separate subclone. Separate molecular signaling schemes for these situations are depicted in Figure 6, illustrating the effect on the promoter of the gene *POMC* (whose protein product is processed to become ACTH, which is then secreted). It is intriguing to consider the possibility that loss of *RASD1* function and mutation of *USP8* may be additive or synergistic in relation to the pathophysiology of CD (Fig. 6D). However, since we did not carry out experimental validation of *RASD1* p.K34M function for this study, we must caution that our claims regarding its involvement in disease biology are only hypothetical and based mainly on what is known about *RASD1* biology from prior studies. Further studies should investigate the functional significance of this novel *RASD1* mutation in the pathogenesis of ACTH tumors.

## METHODS

### WES and Targeted Validation

Paired-end (2×100 bp) WES on Illumina HiSeq 2500 (Illumina) and targeted amplicon validation on PacBio RSII (Pacific Biosciences) was carried as previously described (Uzilov et al. 2016), with the following modifications. For WES, the SureSelect Human All Exon V5 hybridization capture system (Agilent) was used. Libraries from three tumor and nine normal samples were multiplexed in a 2:1 tumor:normal ratio and sequenced on all eight lanes of a High Output flow cell; only two of these samples are presented in this work (others to be published in a future work).

### Variant Calling

WES FASTQ files from the normal and tumor sample were combined into a patient-specific “cohort” and run through an in-house pipeline (Linderman et al. 2014) to yield binary alignment (BAM) and variant call format (VCF) files with germline and somatic variant calls (SNVs and small indels). Briefly, this in-house pipeline implements the Genome Analysis Toolkit (GATK) (McKenna et al. 2010) version 3.2, best practices for alignment, base quality recalibration, variant calling (using HaplotypeCaller), and variant quality score recalibration (VQSR) (DePristo et al. 2011; Van der Auwera et al. 2013). For read alignment, the hg19 human genome reference from UCSC (Rosenbloom et al. 2015) was used. VQSR was set to 99.5% sensitivity. Read pairs whose 5′ coordinates were identical were marked (except for one read pair) as duplicates by the Picard software (http://broadinstitute.github.io/picard) and were not used for variant calling, per the above best practices, to ensure that evidence for each variant was derived from distinct DNA molecules, thus avoiding overcounting and possibly overamplified or oversampled DNA. A GATK genomic interval list was created from the design file from the WES hybridization-capture kit manufacturer; sequencing depth (Table 1) was computed only within these genomic intervals, whereas variant calling was done within these genomic intervals padded by 100 nt on both sides. For somatic variant calling, MuTect (Cibulskis et al. 2013) (version 1.1.6-10b1ba92, HC+PON mode with default settings, using COSMIC [Forbes et al. 2015] version 68, dbSNP [Sherry et al. 2001] version 138, and variant calls from patient-matched normal control as the “panel of normals” setting) and Varscan2 (Koboldt et al. 2012) (version 2.3.5, with flags --tumor-purity 0.5 and --min-var-freq 0.07, then filtered using VS_SPVAL threshold of 20) were used.

SNV calls from Mutect (*N* = 158) and indel calls from Varscan2 (*N* = 97) were loaded into a custom MySQL (Percona MySQL Server Community Edition 5.6.14-rel62.0.483.rhel6) database schema using in-house scripts and annotated using RVS (Hakenberg et al. 2016) and SnpEff 4.0b (Cingolani et al. 2012) using the Ensembl (Aken et al. 2016) version 75/GRCh37 resource bundle. Somatic calls whose population allele frequency in Exome Aggregation Consortium (ExAC) (Lek et al. 2016) exceeded 1% were discarded on the presumption that they are any combination of contamination, a variant present but missed in the normal sample, a low-level artifact could not be pathogenic because it was too common in general population. All remaining SNV (*N* = 152) and indel (*N* = 64) calls were manually reviewed in IGV (Robinson et al. 2011; Thorvaldsdóttir et al. 2013) and the UCSC Genome Browser (Rosenbloom et al. 2015) to inspect supporting alignment quality in the BAM files and mappability of the genomic region in the hg19 human genome assembly, paying attention to whether a variant call was located in a short tandem repeat or a low-complexity sequence region (Benson 1999), a region with self-homology/duplication in the reference genome, or a region of low alignability according to the GEM track from ENCODE/CRG (Derrien et al. 2012). Uncertain calls, many of which were due to a low-level C>A substitution artifact also present in the normal or due to artifacts in padding regions, were manually rejected at this step, resulting in a final list of 25 SNV and 11 indel calls, which are shown in Figure 3, the protein-altering subset of which is shown in Table 2.

### Protein Sequences and Multiple Sequence Alignment

Protein sequences are from UniProt (The UniProt Consortium et al. 2015) (retrieved 2016-09-23); only human sequences selected by UniProtKB curators as canonical protein isoforms were used. HGNC gene symbols, UniProt accessions/isoform identifiers, and RefSeq accessions are as follows:

*RASD1*, Q9Y272,-1 NP_057168.1
*RASD2*, Q96D21-1, NP_055125.2
*DIRAS1*, O95057-1, NP_660156.1
*DIRAS2*, Q96HU8-1, NP_060064.2
*NRAS*, P01111-1, NP_002515.1
*HRAS*, P01112-1, NP_005334.1 and NP_001123914.1
*KRAS*, P01116-1, NP_203524.1

The multiple sequence alignment for Figure 4 was made using the European Molecular Biology Laboratory European Bioinformatics Institute (EMBL-EBI) Clustal Omega web tool (Goujon et al. 2010; Sievers et al. 2011) (http://www.ebi.ac.uk/Tools/msa/clustalo/, used 2016-09-24, default settings) and edited using Unipro UGENE v1.24.2 (Okonechnikov et al. 2012). Protein sequence identity of the RASD subfamily was determined by *blastp* of *RASD1* and *RASD2* against all human proteins in the RefSeq protein database (Altschul et al. 1997, 2005) (http://blast.ncbi.nlm.nih.gov, used 2016-09-25).

## ADDITIONAL INFORMATION

### Data Deposition and Access

All somatic mutation calls passing manual review (including those predicted to not alter protein) are provided as Supplemental File 1 and have been submitted (COSP42647) to the COSMIC database (Forbes et al. 2015). Consent could not be obtained for public release of raw sequencing data.

### Ethics Statement

The study was approved by the Institutional Review Board at the Mount Sinai Medical Center. The patient gave written informed consent before participation, including permission to publish the results.

### Acknowledgments

This work was supported in part through the computational resources and staff expertise provided by the Department of Scientific Computing at the Icahn School of Medicine at Mount Sinai. We thank the Icahn School of Medicine at Mount Sinai (ISMMS) Biorepository and Pathology Core for their support. We thank Andrew F. Stewart, Azad Gucwa, and Wei Ding for helpful discussions.

### Author Contributions

A.V.U., M.Y.F., C.P., and A.S.M. carried out the data processing and analysis. A.V.U., K.C.C., M.Y.F., and E.B.G. wrote the manuscript. K.C.C., Y.L., C.Y.L., M.Z., and A.G. carried out clinical and research coordination. L.C.N. and G.D. carried out the PacBio validation work. M.H. and M.F. carried out the histopathology work. Y.Ki. and M.J.D. carried out tissue processing at the ISMMS Biorepository. K.D.P. carried out surgical resection. Y.Ka., M.M., and R.S. directed the sequencing operations. D.S., E.E.S., R.S., R.C., and E.B.G. directed the study. All authors read and approved the manuscript.

### Funding

This study was supported by an investigator initiated grant from Novartis Pharmaceuticals Corporation to E.B.G.

### Competing Interest Statement

Y.L. is presently an employee of Novartis Pharmaceuticals Corporation. She was employed by Mount Sinai during the course of the work for this manuscript.

### Referees

Masayuki Komada

Anonymous

## Supplementary Material

Supplemental Material
