# Supplementary material for: Identification of a novel RASD1 somatic mutation in a USP8-mutated corticotroph adenoma
Source: Cold Spring Harb Mol Case Stud. 2017 May;3(3):a001602. doi: 10.1101/mcs.a001602 (PMC5411693; doi:10.1101/mcs.a001602)
Supplement: Supplemental Material [file supp_3_3_a001602__index.html]

Identification of a novel RASD1 somatic mutation in a USP8-mutated corticotroph adenoma — Supplemental Material 

# Identification of a novel *RASD1* somatic mutation in a *USP8*-mutated corticotroph adenoma

## Supplemental Material

- Supplemental\_File\_1.vcf.zip
- Supplemental\_File\_1\_Legend.docx
