# Supplementary material for: Identification of a novel RASD1 somatic mutation in a USP8-mutated corticotroph adenoma
Source: Cold Spring Harb Mol Case Stud. 2017 May;3(3):a001602. doi: 10.1101/mcs.a001602 (PMC5411693; doi:10.1101/mcs.a001602)
Supplement: Supplemental Material [file supp_mcs.a001602_Supplemental_File_1_Legend_Refs.docx]

# Supplementary File Caption

## Supplementary File 1

Variant Call Format (VCF) file containing all 36 somatic mutation calls passing manual review. Column “ID” contains the reversible, globally unique identifier (“vkey”) for the alternate allele, as used by RVS (Hakenberg et al. 2016) and DIVAS (Cheng et al. 2015) databases.

## References

Cheng WY, Hakenberg J, Li SD, Chen R (2015) DIVAS: A centralized genetic variant repository representing 150 000 individuals from multiple disease cohorts. Bioinformatics 32:151–153. doi: 10.1093/bioinformatics/btv511

Hakenberg J, Cheng WY, Thomas P, Wang YC, Uzilov AV, Chen R. (2016) Integrating 400 million variants from 80,000 human samples with extensive annotations: towards a knowledge base to analyze disease cohorts. BMC Bioinformatics 17:24. doi: 10.1186/s12859-015-0865-9
